# Environmental factors influencing participation of stroke survivors in a Western Cape setting

**DOI:** 10.4102/ajod.v4i1.198

**Published:** 2015-10-30

**Authors:** Judy Cawood, Surona Visagie

**Affiliations:** 1 Centre for Rehabilitation studies, Stellenbosch University, South Africa

## Abstract

**Background:**

Environmental factors compound or diminish the effects of impairments; therefore they have a direct influence on participation of stroke survivors.

**Objectives:**

To determine environmental barriers and facilitators to participation experienced by a group of stroke survivors in the Western Cape province of South Africa.

**Methods:**

A descriptive, mixed methods study was conducted in 2011. Quantitative data was collected with the *International Classification for Functioning, Disability and Health* core set for stroke (environmental factors), from 53 stroke survivors, sampled through proportional, stratified, random sampling. Data is presented through graphs and tables. Qualitative data was collected from five purposively sampled participants and thematically analysed.

**Results:**

Under products and technology, participants regarded assets, food, products and technology for daily living, transportation, mobility and communication, and access to buildings as barriers. The physical geography and attitudes of friends and society created further barriers. With regard to services, systems and policies - housing, communication, transport and social services created barriers. Health services, as well as support from health care service providers and family were considered facilitators.

**Conclusion:**

A lack of assets compounded barriers with regard to food, products for daily use, communication and transport. Barriers to participation were exacerbated by a lack of services, systems and implementation of policies focused on the inclusion of people with disabilities, as well as minimal access to assistive devices. Recommendations include provision of assistive devices, structural changes to houses, yards, roads and buildings, lobbying for accessible, affordable public transport, access audits of public buildings, and inclusion of non-governmental organisations and home-based care services in a seamless network of care.

## Introduction

Stroke is the 5th leading cause of death in South Africa and in the Western Cape province. It accounts for 5% of deaths in the Western Cape province and 3.5% of deaths nationally (StatsSA 2011). Stroke is also associated with high levels of morbidity. Stroke survivors often experience residual impairments, such as weakness, paralysis, spasticity, perceptual, cognitive, speech and language problems. These impairments can impact negatively on their function and participation in life roles (Gillen 2011; Tipping 2008). Impairments are but one component of a complex, dynamic system that impacts on participation and how disability is experienced (Bickenbach 2009; Shakespeare 2014). Environmental factors can compound or diminish the impact of impairments and activity limitations on life roles (Kostanjsek 2011; Schneidert et al. 2003; World Health Organisation 2001).

The *International Classification of Functioning, Disability and Health* (ICF) provides a framework for exploration of the interaction between environmental factors, the health condition, impairments, activity limitations, and their impact on participation (WHO 2001). According to the ICF: ‘Environmental factors make up the physical, social and attitudinal environment in which people live and conduct their lives’ (WHO 2001:171).

Environmental factors are grouped in five domains:

- products and technology
- natural environment and human-made changes to the environment
- support and relationships
- attitudes
- services, systems and policies (WHO 2001:173–207).

Knowledge and understanding of environmental facilitators and barriers experienced by various populations around the world can assist policymakers, service providers and people with disabilities in promoting greater participation through utilising existing facilitators, and addressing and minimising barriers (Stark 2001). This paper describes environmental barriers and facilitators to participation experienced by a group of stroke survivors in the Western Cape province of South Africa. These stroke survivors were amongst the 71% of the Western Cape province’s population that access public health care services (Day & Gray 2013).

## Methods

**FIGURE 1:**
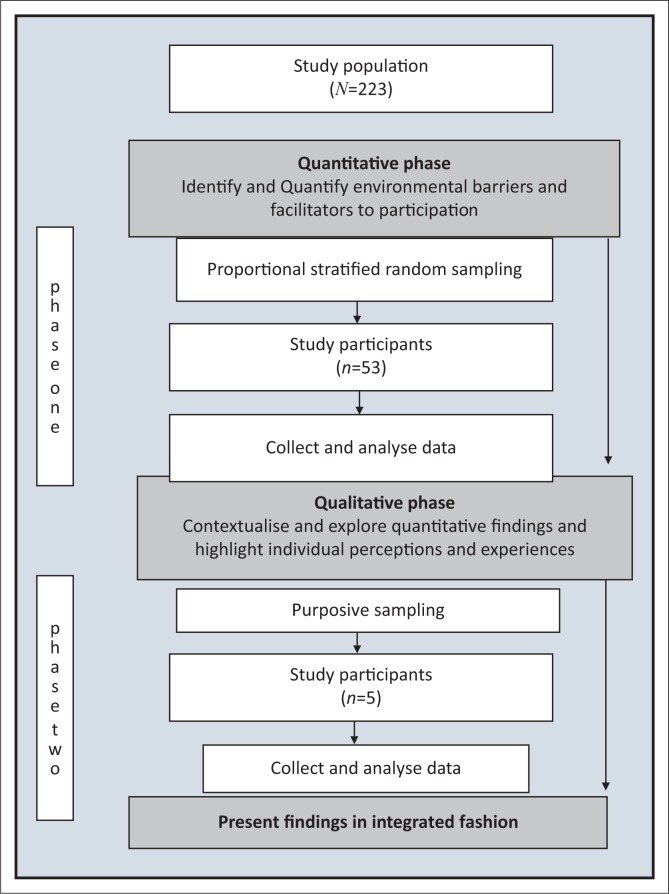
Diagrammatic presentation of the mixed method design with sequential explanatory strategy.

This paper presents some of the results of a descriptive, mixed methods study that used a sequential explanatory strategy, that is, qualitative methods were used secondary to quantitative methods to contextualise and explore quantitative findings, and to highlight individual perceptions and experiences as described by Kroll, Neri & Miller (2005) and presented in Figure 1. The study was conducted in part of the eastern, subdistrict of the Western Cape Metropole Health District. The study population comprised the 223 people older than 18, residing in the study setting, who had experienced a stroke between 1 January 2009 and 31 December 2010, and had accessed public health care services. They were identified from records from hospitals, community health centres (CHCs), and non-governmental organisations (NGOs).

A total of 53 participants were selected to participate in the quantitative phase through proportional, stratified, random sampling (Joubert & Katzenellenbogen 2007). After consultation with a statistician, the primary author decided that approximately 50 participants would allow completion of the study within a reasonable period (extensive data was collected), while ensuring a sample large enough to allow statistical analysis of data. The five geographical subunits in the study setting were used as strata to ensure that participants from all communities in the study setting were included. The names of stroke survivors in each stratum were listed alphabetically and numbered. A computer programme was used to generate random numbers to select study participants. The number of participants from each stratum was proportional to the total number of names in each.

Data was collected between March and August 2011 by an occupational therapist. Quantitative data was collected with a demographic data coding form and the ICF’s core set for stroke (environmental factors). The latter was developed by 39 experts from 12 countries through a systematic review of the literature, a Delphi exercise and empirical data collection. It contains 33 categories from the five domains of environmental factors, deemed important to most stroke survivors (Geyh et al. 2004). Scoring options range from -4 through 0 to 4, where -4 denotes a complete barrier, -3 a severe barrier, -2 a moderate barrier and -1 a mild barrier. A score of 0 indicates that the aspect has no influence on the person’s life. A score of 4 denotes a complete facilitator, 3 a substantial facilitator, 2 a moderate facilitator and 1 a mild facilitator (WHO 2001). During data collection, the same examples were given to all participants to help them understand the question without creating bias.

The aim of the qualitative phase was to seek explanations that could enhance understanding of the quantitative figures (Silverman 2013). A heterogeneous group of five participants was purposively sampled for the qualitative phase: two female and three male participants, with ages ranging from 44–69 years. They had varying functional ability - from needing assistance with all activities to being totally independent. Qualitative data was collected through semi-structured interviews guided by an interview schedule. Quantitative and qualitative data was collected on separate occasions. Depending on the participant, this was done at the participant’s home or at a central venue. Transport was provided for travelling to the central venue.

Quantitative data was entered onto a spreadsheet from which graphs and tables were drawn. No further statistical analyses were done for this descriptive paper since it was not the purpose to explore causal relationships. Qualitative data was analysed according to themes that were predetermined from quantitative findings.

### 

#### Ethical considerations

Participation in the study was voluntary and written informed consent was obtained from every participant. Special consideration was given to determining the competence of the stroke survivor to make an informed decision about participation in the study. In instances where the person was not able to give consent, an authorised caregiver gave consent. Where proxy consent was given, an indication was obtained from the stroke survivor as to their willingness to participate. Participants were assured that all personal information that could divulge their identity would be kept confidential. Approval for this study was obtained from the Committee for Human Research at Stellenbosch University. Permission to acquire the names and contact details of people who had a stroke was obtained from the Western Cape Department of Health, the relevant points of service delivery and NGOs.

## Results

The majority of participants (66%) were between 51 and 70 years old (Table 1).

**TABLE 1a:** Demographic information of participants (*n* = 53). (Percentages have been rounded).

Age	%	Education	%
20–30	2	No formal education	11
31–40	4	Grade 1–3	11
41–50	6	Grade 4–7	34
51–60	28	Grade 8–11	30
61–70	38	Grade 12	8
71–80	19	Tertiary education	6
80+	4	-	-

**TABLE 1B:** Demographic information of participants (*n* = 53). (Percentages have been rounded).

Income of family unit	%	Gender	%
No income	6	Men	55
R1 – R2000.00	30	Women	45
R2001.00 – R5000.00	53	-	-
R5000.00+	11	-	-

### Products and technology

**FIGURE 2:**
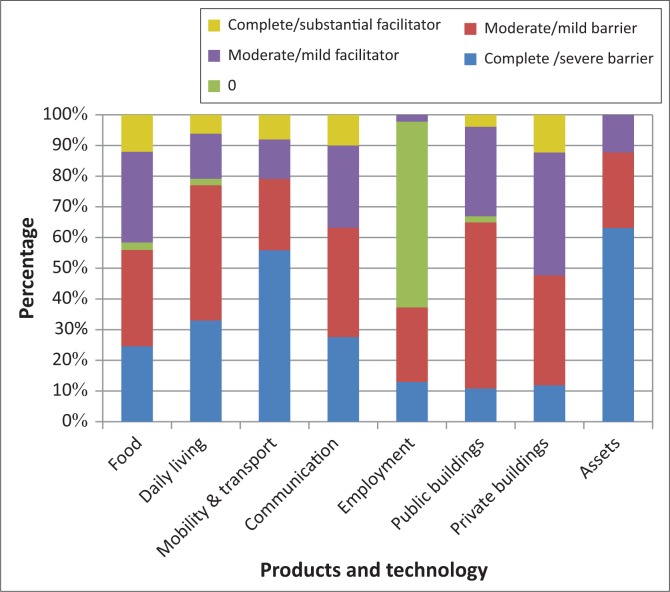
Products and technology experienced as barriers and facilitators (*n* = 53).

All but two aspects related to products and technology created barriers to participation for more than 50% of participants (Figure 2). Almost all participants (89%) indicated a lack of assets (defined by WHO 2001:181 as ‘Products of economic exchange, such as money, goods, property and other valuables that an individual owns or of which he or she has rights of use’) as a barrier. Participants indicated that often they could not afford rent, telephone services or food.

Overall, 77% of participants identified products and technology for personal use in daily living (e.g. clothing, household appliances, furniture and assistive devices) as a barrier. Mobility assistive devices were the category of assistive devices most frequently issued to participants (Table 2).

**TABLE 2:** Assistive devices received by participants (*n* = 53).

Mobility assistive devices	Percentage of participants who received the device (%)	Other assistive devices	Percentage of participants who received the device (%)
Wheelchair	53	Tray table	6
Cane / crutch	53	Bath board	2
Walking frame	15	Adapted kitchen utensils	2
Ankle foot orthosis	2	-	-

A lack of transport created barriers to community participation and accessing services for 80% of participants. The wife of a 54-year-old participant explains: ‘it is so difficult to get to the hospital. I have to ask my son; often he has to put in leave at work to help’. Those who could use taxis also experienced challenges. Some found the distance from the taxi rank to the hospital too far to walk. A 65-year-old male participant described the taxi drivers as ‘impatient and in a hurry’ and unwilling to help people who needed assistance to embark. Although Health Net Services (state-provided transport between health care service facilities) are available, some participants were unaware of this service or how to access it. Others reported difficulty in getting to the primary health care service facilities from where this transport departs.

There were 38% of participants that were dependent on friends and family for transportation, and 32% had to pay for this ‘service’ (Table 3).

**TABLE 3:** Transport used by participants (*n* = 53).

Type of transport	% of participants
Taxi	30
Bus/train	0
Relatives and friends	38 (32% paid for this service)
Own transport	15 (4% drove self)
No transport	17

In the absence of transport, participants walked or used wheelchairs to access the community. Wheelchair users found it difficult to use taxis, which increased transport costs for them: for instance, it cost R100.00 ($8.33) to hire a car for a trip that would cost R14.00 ($1.17) by taxi. Several participants commented that flights of stairs at stations in the area created barriers to using trains.

Products and technology for communication were seen as a barrier by 64% of participants (Figure 2). This mainly took the form of being unable to afford phone services or airtime. Most participants (94%) had access to a television in their homes, but only 4% had access to computers and email. The majority (60%) of participants were of the opinion that products and technology related to employment were neither a barrier nor facilitator; this was mainly because they were past retirement age (Figure 2).

Participants found the question on design of public buildings difficult to answer, as the majority only left their homes to access health care services or to collect their pension, and these buildings were accessible. Overall, 65% rated public buildings in the study setting as being inaccessible, mainly because of steps. One participant, a wheelchair user and avid soccer fan, described how four of his friends collected him every weekend to support club matches. If they needed to get up steps in the stadium, they picked him up in his wheelchair or got other people in the crowd to assist.

Just over half (53%) of participants considered their homes to be a facilitator, while 48% indicated that steps/stairs, a lack of facilities such as indoor toilets and the size and structure of their homes created barriers (Figure 2).

### Natural environment and human-made changes to the environment

The geography of their surroundings was perceived as a barrier by 71% of participants. They commented on the difficulty of walking or pushing wheelchairs through sandy soil, over uneven terrain and through potholes. Pavements were often lacking or uneven. Loose sand (51%) or hard uneven soil (38%) covered most yards, making it difficult to manoeuvre wheelchairs or walk, particularly in the light of poor balance and sensory impairments.

### Support and relationships

**FIGURE 3:**
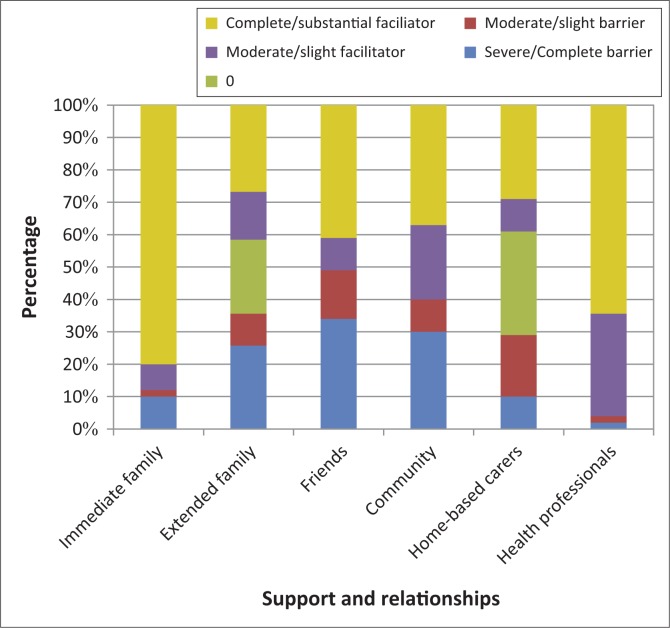
Support and relationships experienced as barriers and facilitators (*n* = 53).

A positive finding was that 88% of participants felt their immediate family was supportive (Figure 3). Extended family provided less support. For 23% of participants, a lack of transport and finances prevented contact with extended family living elsewhere. The majority (60%) of participants found acquaintances in the community supportive. During qualitative interviews, participants indicated that due to physical weakness they felt vulnerable and feared being attacked by ‘skollies’ (criminals) or ‘tik gangs’ (drug abusers) in the community.

Home-based care services (personal care providers) were limited and 61% of participants did not receive home-based care - a lack that 29% of participants saw as a barrier. Participants and caregivers showed appreciation of services rendered by health professionals, and 97% of the participants saw assistance by health professionals as a facilitator (Figure 3).

### Attitudes

**FIGURE 4:**
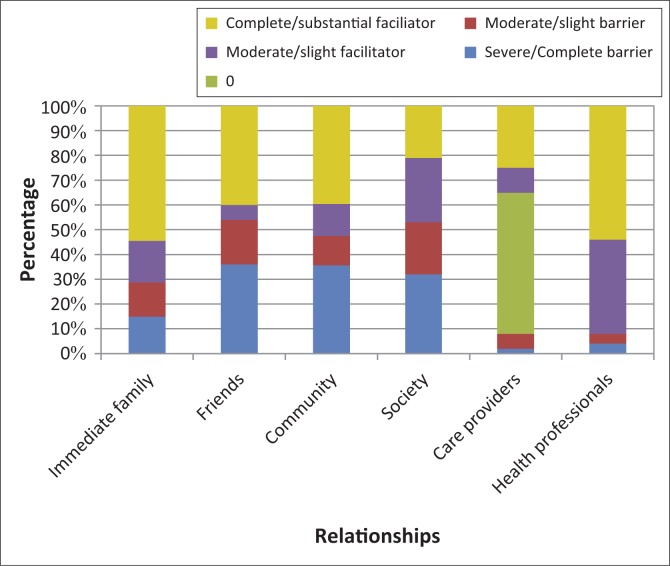
Attitudes experienced as barriers and facilitators (*n* = 53).

The majority of participants (72%) found the attitudes of their immediate family positive (Figure 4). However, some participants felt that while family members cared for them, they did it reluctantly and from a sense of duty.

The attitudes of community members were regarded a barrier by 47% of participants, and 53% perceived societal attitudes as a barrier. A 79-year-old female stroke survivor reported that people regarded her as ‘mad and incompetent’ after the stroke, which she found extremely hurtful. A 46-year-old male stroke survivor said that after shaking his hand, people would wipe their hands on their own clothing, as if they had touched something contaminated.

A small minority of participants (8%) regarded the attitudes of home-based carers (personal care providers) as negative, and were concerned about carers sharing confidential information about their clients with other people. Some found them unreliable and reported that they did not always keep appointments. The attitudes of health professionals were seen as a facilitator by 92% of participants (Figure 4).

### Services, systems and policies

**FIGURE 5:**
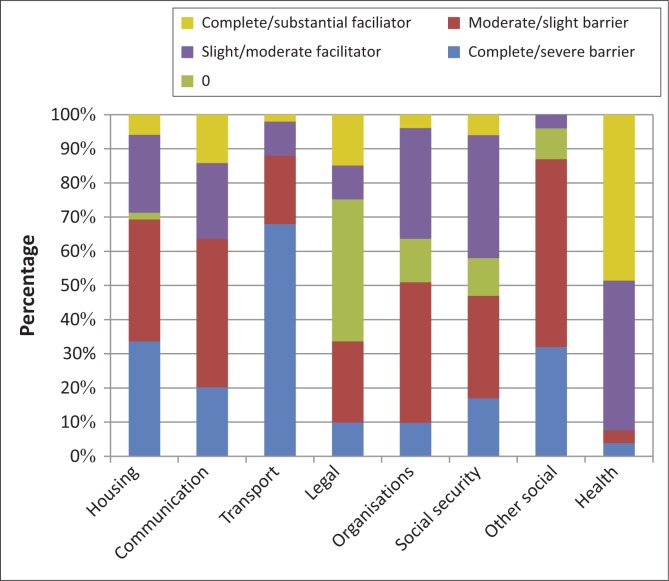
Services, systems and policies experienced as barriers and facilitators (*n* = 53).

The majority of participants (70%) regarded housing services as a problem (Figure 5). They expressed concerns about the lack of housing, the small size and poor quality of government subsidised houses, and the difficulty of getting repairs done. Communication services was regarded as a barrier by 63% of participants because of the costs of airtime and landlines, as well as a lack of public telephones and facilities for sending faxes and email. Similarly, transport services were regarded as a barrier by most (88%) participants (Figure 5). A 60-year-old male participant who was asked what the greatest need of stroke survivors was replied: ‘Transport’.

Nearly half (47%) of the participants indicated that they did not receive assistance from organisations and associations. Others mentioned help from a stroke support group (15%), BADISA (9%), the society for the aged (6%), and other community and religious organisations. The stroke support group has nine subgroups running in the various communities. Those who attend its activities found it supportive and educating:

‘Aah!! It means so much to her. Before she gets fetched on a Monday, she is so excited. She will be dressed early and be ready and waiting. Then she will tell me what happened that day’. (Husband of 45-year-old participant)

‘And then I read in the newspaper about the stroke support group. That is where I learned the most about stroke. Nobody else explained to me. You interact with others. This is when I really started to understand what everything was about’. (60-year-old male participant)

‘You speak to other people who have had the same experience or are worse off than you. I think suddenly my eyes were opened’. (60-year-old male participant)

Participants had ambivalent feelings about social security services, systems and policies. The regular income was seen as a facilitator by 42% of participants (Figure 5). On the other hand, the small amount of money was considered a barrier by 47%, as were the difficulties participants encountered to register for disability grants. Specific complaints included:

- Long queues at registration, and people being sent away and told to come back on another day.
- Unfairness: ‘He knows Piet, Paul and Klaas; he knows them. Let them through and the rest of us have to stand in the queue’ (husband of 45-year-old participant).
- Delays of several months where the survivor was without income while the paper work was being processed after a temporary disability grant expired.
- Difficulties in securing a doctor’s appointment for completion of the medical forms.
- Fear of lodging complaints. Participants feared reprisal and that they would not be assisted.

The lack of general social support services and systems (e.g. assistance with activities of daily living, shopping, transport and housework) were identified as a barrier by 87% of participants (Figure 5). The need for assistance in this regard is summed up by this participant:

‘It is like a prison sentence. I am actually a prisoner. I want to go to Checkers. Please take me quickly. Checkers is in five minutes walking distance from our house. It is a huge problem. I have to sit and plan how I can get to Checkers’. (60-year-old male participant)

In another example, a woman’s 14-year-old son had to take off school to collect her medication and take her to the clinic for appointments.

With regard to health care services, participants complained about long queues and waiting times:

‘There are just rows and rows of people that sit there’. (60-year-old male participant)

‘If you go to the clinic, you have to be very patient. What I can’t understand is why people have to be there so early and then are only helped in the afternoon’. (Husband of 45-year-old participant)

‘Usually when I go, I leave the house at 6am or 6.30am. I then get back at about 4.30pm. That is how long I have to sit and wait’. (44-year-old female participant)

Participants complained about the noise level in waiting rooms and that they could not hear their numbers being called - this often meant they missed their turns.

Despite these complaints, participants were positive about health care providers and services, as shown in Figures 3–5. Participants acknowledged the shortage of human resources and its effect on service delivery:

‘One must just accept that if you go to the clinic you must take a bag of patience with you!’ (Husband of 45-year-old participant)

‘I am not pointing fingers at the doctors, because I think all the doctors are overworked. If you look there, there are hordes of people. It is a hell of a task for them’. (60-year-old male participant)

‘It doesn’t help to be impatient … you must always think about the person helping you. That person has also got things they have to do. If a patient complains and carries on, that person is not going to want to help you. That is why I am always relaxed. I keep calm, because I also consider that person. He has got lots of people he has to help and he is only one person. I am patient, I wait for my turn’. (44-year-old female participant)

Examples of poor service delivery were mentioned, including lying in urine, being assisted by fellow patients to get to the toilet because nurses were unavailable, dealing with intoxicated service providers, and sustaining a leg injury while being moved in hospital that allegedly resulted in an amputation. Challenges caused by scope of practice and lack of a stroke protocol were also mentioned:

‘She [*professional nurse*] knew exactly what to do, but she waited for instructions. This is something that can result in a patient’s condition deteriorating … if the sister knows she must put up the drip and that it must be put up now. Another thought that comes to mind is why is no doctor at the local hospital able to help with a stroke patient in the beginning stage as it starts to take place? Surely for the trained eye the symptoms must be known?’ (Husband of 45-year-old participant)

With regard to municipal services, all participants had access to electrical power, and all but one had access to running water in their homes. However, free quotas of water were finished quickly in large households and participants did not have the finances to pay for additional water. One caregiver whose husband was bedridden and incontinent reported how difficult it was to keep the linen clean with the limited amount of free water. Some participants complained about dogs roaming the streets being a barrier to participation: with their poor walking balance they felt vulnerable. These participants were not aware that municipal by-laws prohibit dogs from being on the street unless they are on a lead.

## Discussion

A lack of assets, as well as transport and mobility created a barrier to participation for the majority of participants. Barriers with regard to transport are highlighted both under products and technology, and services, systems and policies. Other factors that created barriers to more than 50% of participants included food, products and technology for daily living and communication, access to public buildings, physical geography, attitudes of friends and society, as well as the following services, systems and policies: architecture and construction, housing, communication, associations, and general social support. These findings were similar to those reported by Maart et al. (2007) with regard to public buildings and housing, but contrasted in other respects. Maart et al. (2007) found that the climate, labour and education services also created barriers to participation in the Western Cape province. The discrepancies might be due to methodological differences, such as different study populations and the use of a different ICF environmental factors tool.

In the category of ‘products and technology’, participants experienced more barriers than facilitators overall – largely because of a lack of assets. Assets include money, goods (such as property), as well as skills and knowledge that can be used to earn money (WHO 2001). Participants were retired, had lost their ability to earn because of decreased physical and cognitive ability resulting from the stroke, or encountered environmental barriers that prevented them from accessing employment. The lack of assets impacted on access to food, as well as products and technology for daily living, such as furniture and appliances. A number of participants reported a shortage of food and appeared undernourished. In contrast, some participants were overweight. It is recognised that maintaining a correct diet plays an important role in controlling diabetes and hypertension, as well as preventing obesity - three of the risk factors for stroke (Bryer et al. 2010; Mayosi 2013). With regard to products for daily living, furniture (e.g. a sturdy bed with a firm mattress or a table) plays an important role in ensuring optimal positioning post-stroke. Correct positioning, in turn, decreases complications like spasticity, pain and contractures, which all impact negatively on function.

A lack of assets impacts negatively on people’s ability to access assistive devices and adaptations that can enhance function, and leaves them dependent on rehabilitation services for adaptations and assistive devices. The results show that few assistive devices other than mobility devices were issued. Something as simple as a lap tray - which is inexpensive, can improve sitting posture, provide a surface for positioning a paralysed arm and a surface on which to eat in circumstances where people have no table - was provided to only 6% of participants.

One participant had been issued with a bath board to facilitate bath transfers. No other devices - such as grabs rails or plastic chairs for the shower, which can enhance safety during washing - were issued. In a Danish study, Sørensen et al. (2003) found that assistive devices to assist with bathing was provided to 63% of participants, and that grab rails were installed in the homes of all of the participants. Participants in a Swedish study by Randström, Asplund and Svedlund (2012) pointed out the importance of activities such as washing oneself in the promotion of independence and how the provision of assistive devices can assist in achieving this. In the current study (Cawood 2012), the inability of many participants (43%) to use the toilet independently caused strain on caregivers and excessive use of nappies. Considering that 85% of the homes visited had indoor toilets, it is possible that had grab rails been installed, more people could have been independent in toileting.

With retirement, recreational activities can play an increasingly important role in people’s lives. Participants enjoyed reading the newspaper, magazines or their Bible, but were finding this progressively difficult due to a lack of spectacles. At the time of the study, people had to travel approximately 35–50 km (depending on where they were living) for vision testing and spectacles through government services. Participants struggled to access this service. As one 75-year-old male participant said: ‘Either you have an appointment and you don’t have the money, or you have the money and you can’t get an appointment’. A further barrier to reading was a lack of additional assistive devices. A 62-year-old participant, who was an avid reader, lost the function of both upper limbs due to multiple strokes. He was no longer able to turn pages, an action that can be accomplished with various assistive devices.

All participants who could not walk safely indoors had received wheelchairs. This is a positive finding since other South African studies report a lack of access to wheelchairs (Saloojee et al. 2007; Visagie, Scheffler & Schneider 2013). However, other participants probably need a wheelchair for long distances and in situations where terrain was uneven (as described earlier) to ensure community mobility (Greer, Brasure & Wilt 2012). Another assistive device recognised (Gillen 2011) as improving ambulation in stroke survivors with weak dorsiflexion is an ankle foot orthosis (AFO). Only one participant had received an AFO. Considering that 68% of participants had been issued with walking aids, it is probable that more of them would have benefited from an AFO.

Maleka, Stewart and Hale (2012) found community mobility challenging for stroke survivors in a study done in rural Limpopo and urban Gauteng provinces. The current study supports Maleka *et al.*’s findings that the cost of transport was greatly increased because participants could not use taxis and trains. Minibus taxis are the main means of transport for poorer communities in South Africa. As described by other authors, the taxi operators are pressed for time and space (Chakwizira et al. 2010; Kahonde, Mlenzana & Rhoda 2010; Ntamo, Buso & Longo-Mbenza 2013). Thus they might prefer not to stop for someone in a wheelchair since the person takes longer to embark and disembark and the wheelchair occupies the space of an extra passenger. In addition, taxis drive specific routes and participants have to walk to a taxi rank or the route to use the taxi instead of being picked up at home. The results of the current study showed the high cost of hiring private transport, as also demonstrated by Kahonde et al. (2010). In the light of the lack of assets, this might be unaffordable for some.

Another negative consequence of insufficient assets was a lack of access to phone services. Apart from diminishing contact with loved ones, this left participants without a ready means of contacting emergency services. It also creates difficulties for health care services providers to confirm appointments and inform people of pathology results. The need for specialised communication devices amongst persons who had a stroke might be less than for other types of assistive devices (Bouffioulx, Arnould & Thonnard 2011; Sørensen et al. 2003), and sophisticated computerised communication devices are not always effective for aphasic clients with cognitive impairments. However, basic pictographic communication boards can improve communication for those who experience problems with expressing themselves (Howe 2008).

With regard to housing, steps at the front and back door and uneven and sandy terrain created challenges for both wheelchair users and ambulant stroke survivors. In addition, homes were generally small and crowded, and there was insufficient space to manoeuvre wheelchairs. These barriers highlight the importance of therapists obtaining detailed information on the home environment, and engaging with municipal authorities, caregivers and families with regard to the necessity of ramps, paved pathways and the most suitable products to use. Entrances to the homes of 89% of participants in the Danish study by Sørensen et al. (2003) were modified, while adaptations were made in the case of 16% of participants in a Belgium study by Bouffioulx et al. (2011).

It was heartening to find that public buildings often accessed for services by persons with disabilities were accessible. However, the general surroundings, such as road and sidewalk surfaces, require attention. These surfaces should be hard, and free of obstacles such as potholes. Results illustrated how attitudes of participants, friends and society can facilitate participation. But no person should have to suffer the indignity of having to be carried up stairs. Inaccessible public spaces are a major factor contributing to the exclusion of people with disabilities from mainstream society and denying them basic human rights (Banda-Chalwe, Nitz & De Jonge 2012).

Another very positive finding was that both support from and attitudes of immediate family were seen as facilitators by the majority of participants. This finding coincides with findings by Rhoda (2012). However, attitudinal challenges were identified. This might be due to multiple stressors: families might be stressed by the daily struggles caused by a lack of assets. In addition, caregiving duties are physically and emotionally draining and can lead to negative emotional experiences amongst caregivers and what might be seen as negative attitudes. Family carers need respite and time for themselves (Hassan, Visagie & Mji 2011a; Pierce et al. 2007). Thus it is unfortunate that in many instances extended family was not in close enough proximity to provide assistance.

Organisations in the community and home-based carers can play a role in supporting family carers. This was shown by the positive comments on home-based carers and the stroke support group, and the number of participants who saw these services as a facilitator. However, many participants said they did not see how home-based carers could play a role in their circumstances. Others said home-based carers did not visit them and saw this as a barrier. A shortage of home-based care services in the Western Cape province was also identified by Maart & Jelsma (2013) and Hassan, Visagie and Mji (2011b).

Despite limited physiotherapy and occupational therapy – and even less speech therapy – and dietary and psychosocial counselling (Cawood 2012), health care service providers and services were seen as a facilitator by many participants. This finding is similar to that of Kahonde et al. (2010) and Rhoda (2012) from studies done in the Western Cape, but in contrast to other Western Cape findings from Maart and Jelsma (2013) and Namibian findings from Van Rooy et al. (2012). Participants realised the constraints and challenges that health care service providers faced and thus, even though they complained about long waiting times and other challenges, they still felt the service was a facilitator. Strategies such as fast tracking persons with disabilities and pre-packing medication might decrease waiting times.

Participants saw themselves as vulnerable and a soft target for criminals. This is unfortunately a realistic fear given the high levels of violence in the poorer areas of the Western Cape. The impact of unemployment on violence in these locations is illustrated by a participant in a study done by Gibson (2013:4): ‘Violence becomes an outlet. People are unemployed and they rob and hurt other people in order to make a living’. This fear, in conjunction with the transport problems, caused some participants to stay at home most of the time. Maleka et al. (2012) have identified social isolation as a challenge that stroke survivors experience – the current study population might be at risk of social isolation.

The challenges experienced by participants with social services and accessing disability grants are unfortunately not unique to this setting and have been described in other South African settings (Saloojee et al. 2007; Surender et al. 2007). It is especially worrying that participants feared retribution should they complain.

### Study limitations

No power analysis was done to determine a sample size. The sample was too small to allow statistical analysis of data between geographical subunits.

## Recommendations

Environmental barriers are best addressed by multisectoral action. In this instance, government departments (health, housing, transport, social services, law and order), local government, NGOs, communities, and persons with disabilities need to collaborate to address the barriers experienced by study participants. For instance, improved transport for persons with disabilities would mean discussions between elected counsellors, community leaders, people with disabilities, NGOs, taxi consortia, bus service operators, the South African Rail Commuter Corporation and the provincial department of transport.

Similarly, multiple role players – provincial and local government as well as people with disabilities, their families, NGOs, rehabilitation professionals and the police service – should liaise to make communities safe for people with disabilities to leave their homes without fear of being targeted by criminals and stray animals. Different representatives from the same governmental and non-governmental organisations should focus on ensuring cement or tarred sidewalks with kerb cuts to facilitate community mobility. In addition, access audits can determine to what extent inaccessibility of public buildings is a barrier to participation.

Comprehensive assessment for and provision of assistive devices and adaptations to the home environment is part of rehabilitation service provision and must be done by service providers with funding from the Department of Health.

NGOs need to network closely with the Department of Health and health care providers to ensure they are regarded as part of a ‘seamless network’ of services available to stroke survivors. Home-based care services should be extended. There is also a need for further study to explore home-based carers’ adherence to codes of ethical practice.

The Department of Social Services should ensure that social assistance grant applicants should be given an information sheet in their home language, assuring them of their rights and indicating the procedure to follow should they have complaints or queries. The perception of favouritism and retribution in the case of complaints must be explored through further study. Where stroke survivors are issued with a temporary disability grant, social and health care service providers must ensure that they understand the process of re-application and that they know reapplication should be started well before the temporary grant expires.

## Conclusion

Environment barriers - especially with regard to products and technology, and services, systems and policies – hindered participation of stroke survivors in the study setting. A lack of assets compounded barriers with regard to food, products for daily use, communication and transport. Barriers to participation were exacerbated by a lack of services, systems and the implementation of policies focused on the inclusion of people with disabilities, as well as minimal access to assistive devices.
